# Supplementary material for: Merging bioactivity predictions from cell morphology and chemical fingerprint models using similarity to training data
Source: J Cheminform. 2023 Jun 2;15:56. doi: 10.1186/s13321-023-00723-x (PMC10236827; doi:10.1186/s13321-023-00723-x)
Supplement: Supplementary file 5 — Additional file 5: Figure S1. Features used in the similarity-based merger models: a logistic regression model that takes the predicted probabilities from individual models and the test compound’s similarity to the active compounds in the training data in both feature spaces, structural and morphological. Figure S2. Distribution (a) Balanced Accuracy for 171 assays (out of 177 assays) and (b) F1 scores for 177 assays for all models, namely, Cell Painting, structural models, baseline models of soft-voting ensembles, hierarchical models, and the similarity-based merger models. An assay was considered for a paired significance test only if the the balanced accuracy>0.50 and F1 score>0.0 for at least one of the models. Figure S3. (a) Number of assays that were predicted with a Balanced Accuracy above a given threshold. (b) Distribution of assays with Balanced Accuracy > 0.70 common and unique to all models, Cell Painting, Morgan Fingerprints, baseline models of soft-voting ensemble, hierarchical model, and the similarity-based merger models, over 177 assays used in this study. Figure S4. Distribution of AUC Scores for 177 assays used in this study for (a) Cell Painting and Structural Models, (b) Soft-Voting Ensemble and Hierarchical Model, (c) Similarity-based merger model and Hierarchical Model, and (d) Similarity-based merger model and Soft-Voting Ensemble. Any assay above or below the diagonal ($x=y$x=y) line performs better than the other model. Figure S5. Relative improvement (green) or deterioration (red) in performance on using similarity-based merger models compared to soft-voting ensemble methods over the public dataset comprising 162 assays out of 177 assays where either model performed better than a random classifier (A [file 13321_2023_723_MOESM5_ESM.docx]

Supplementary Information

Merging Bioactivity Predictions from Cell Morphology and Chemical Fingerprint Models Using Similarity to Training Data

Srijit Seal^1^, Hongbin Yang^1^, Maria-Anna Trapotsi^1^, Satvik Singh^2^, Jordi Carreras-Puigvert^3^, Ola Spjuth^3,^*, Andreas Bender^1,^*

*^1^* Yusuf Hamied Department of Chemistry, University of Cambridge, Cambridge, United Kingdom

*^2^* Department of Applied Mathematics and Theoretical Physics (DAMTP), University of Cambridge, Cambridge, United Kingdom

*^3^* Department of Pharmaceutical Biosciences and Science for Life Laboratory, Uppsala University, Uppsala, Sweden

* Email: [ab454@cam.ac.uk](mailto:ab454@cam.ac.uk), [ola.spjuth@farmbio.uu.se](mailto:ola.spjuth@farmbio.uu.se)

Machine Learning, Cell Painting, Structure, Toxicity, Bioactivity, Applicability Domain


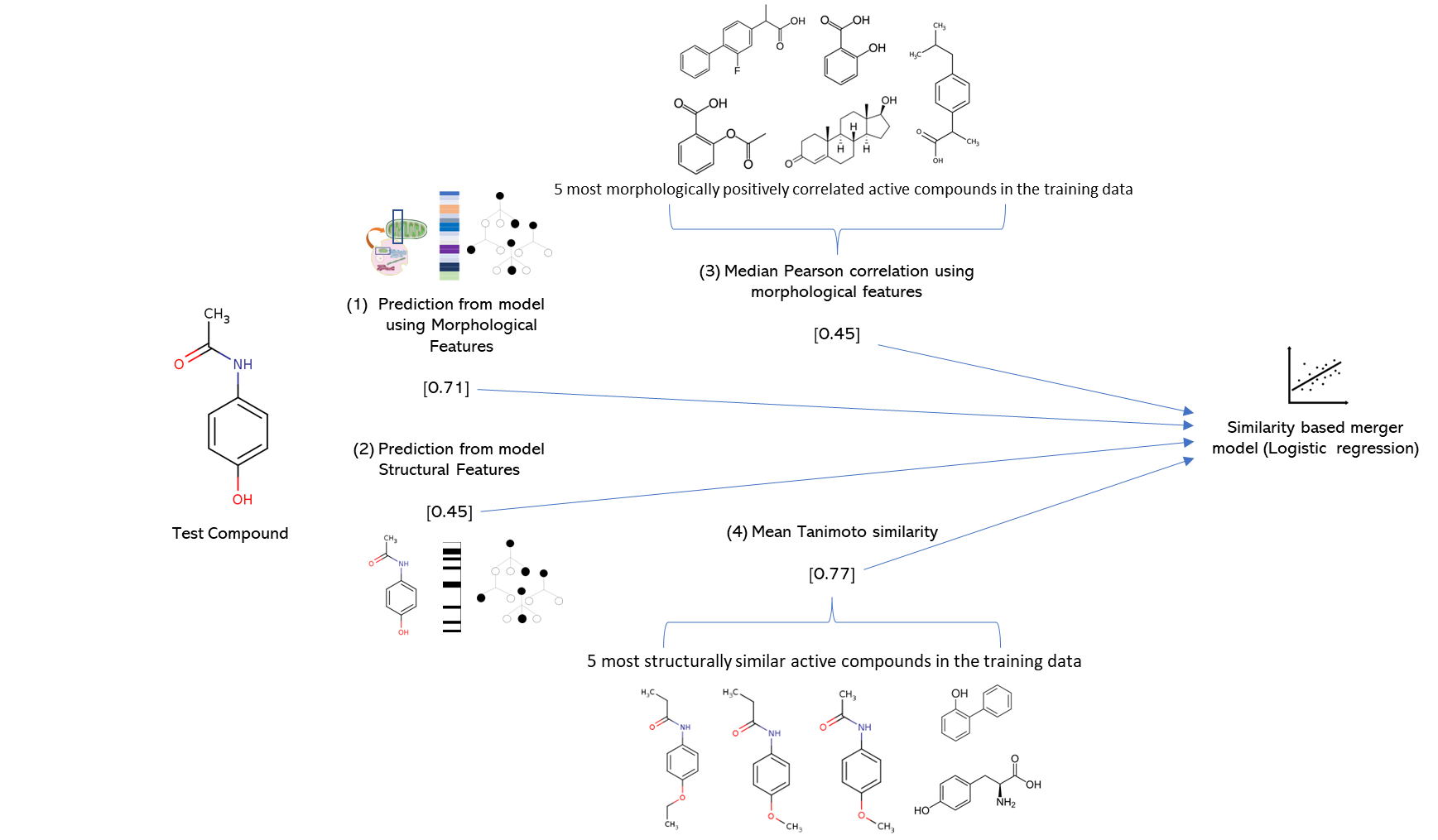


Figure S1: Features used in the similarity-based merger models: a logistic regression model that takes the predicted probabilities from individual models and the test compound’s similarity to the active compounds in the training data in both feature spaces, structural and morphological.


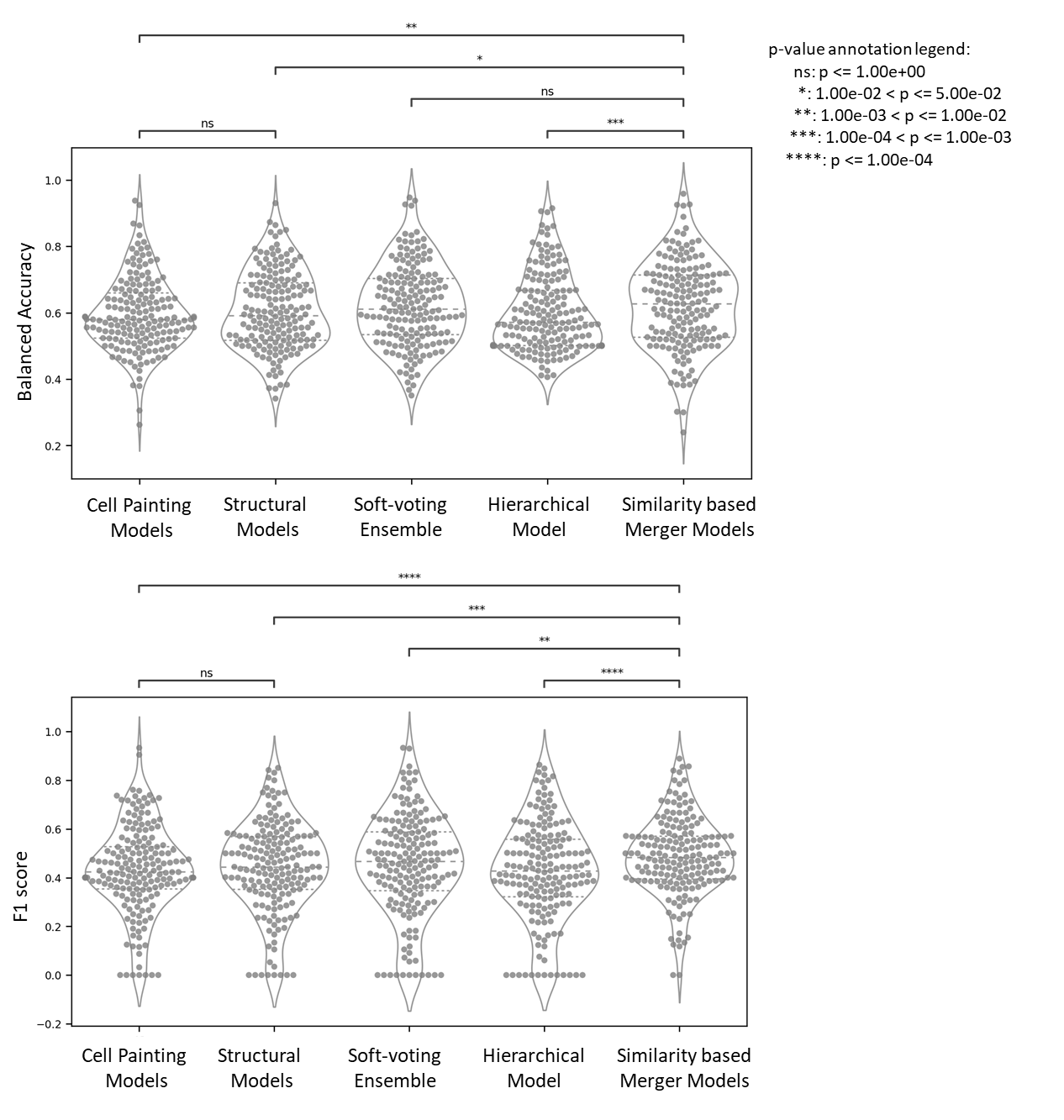


Figure S2: Distribution (a) Balanced Accuracy for 171 assays (out of 177 assays) and (b) F1 scores for 177 assays for all models, namely, Cell Painting, structural models, baseline models of soft-voting ensembles, hierarchical models, and the similarity-based merger models. An assay was considered for a paired significance test only if the the balanced accuracy>0.50 and F1 score>0.0 for at least one of the models.


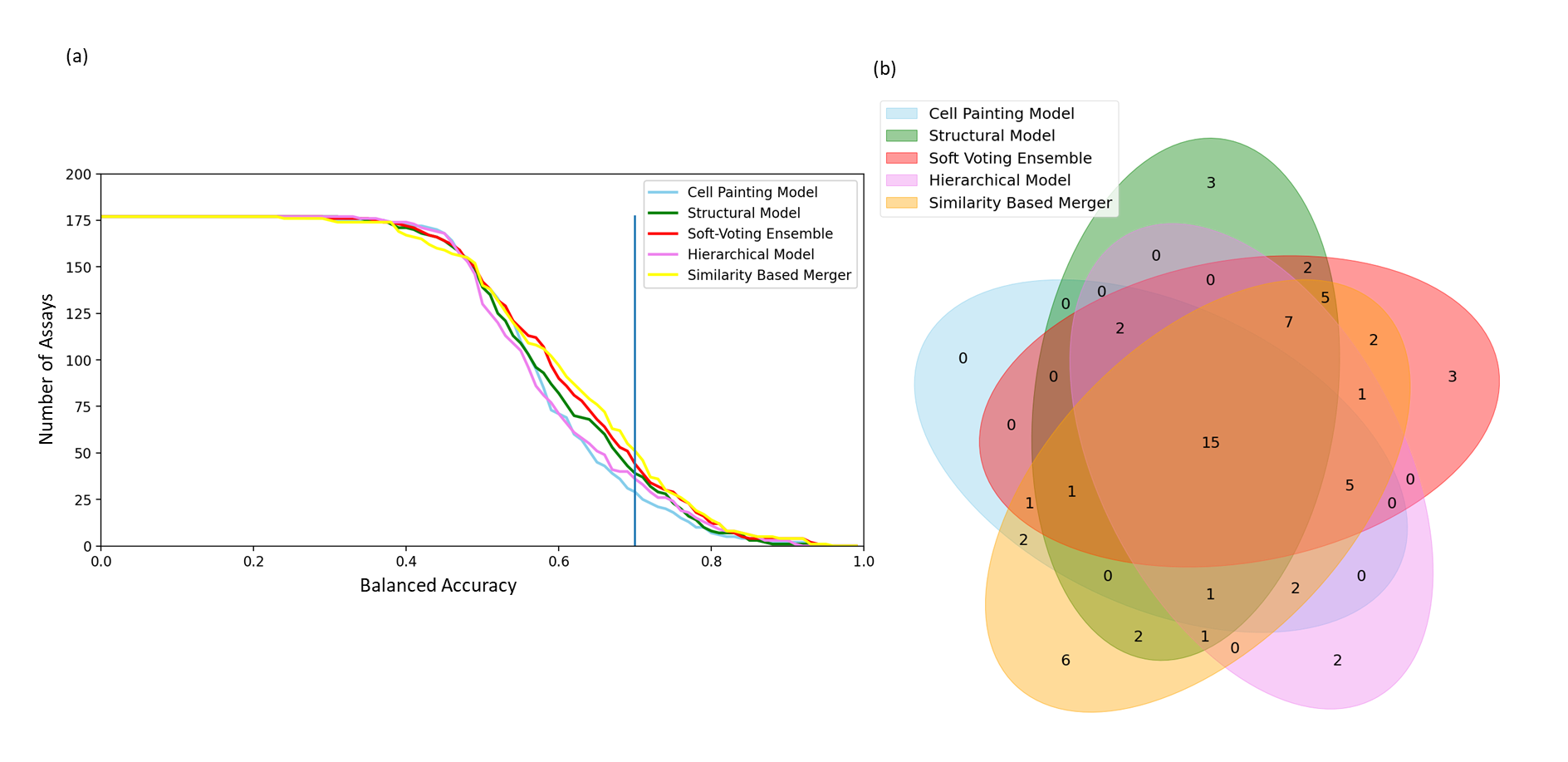


Figure S3: (a) Number of assays that were predicted with a Balanced Accuracy above a given threshold. (b) Distribution of assays with Balanced Accuracy > 0.70 common and unique to all models, Cell Painting, Morgan Fingerprints, baseline models of soft-voting ensemble, hierarchical model, and the similarity-based merger models, over 177 assays used in this study.


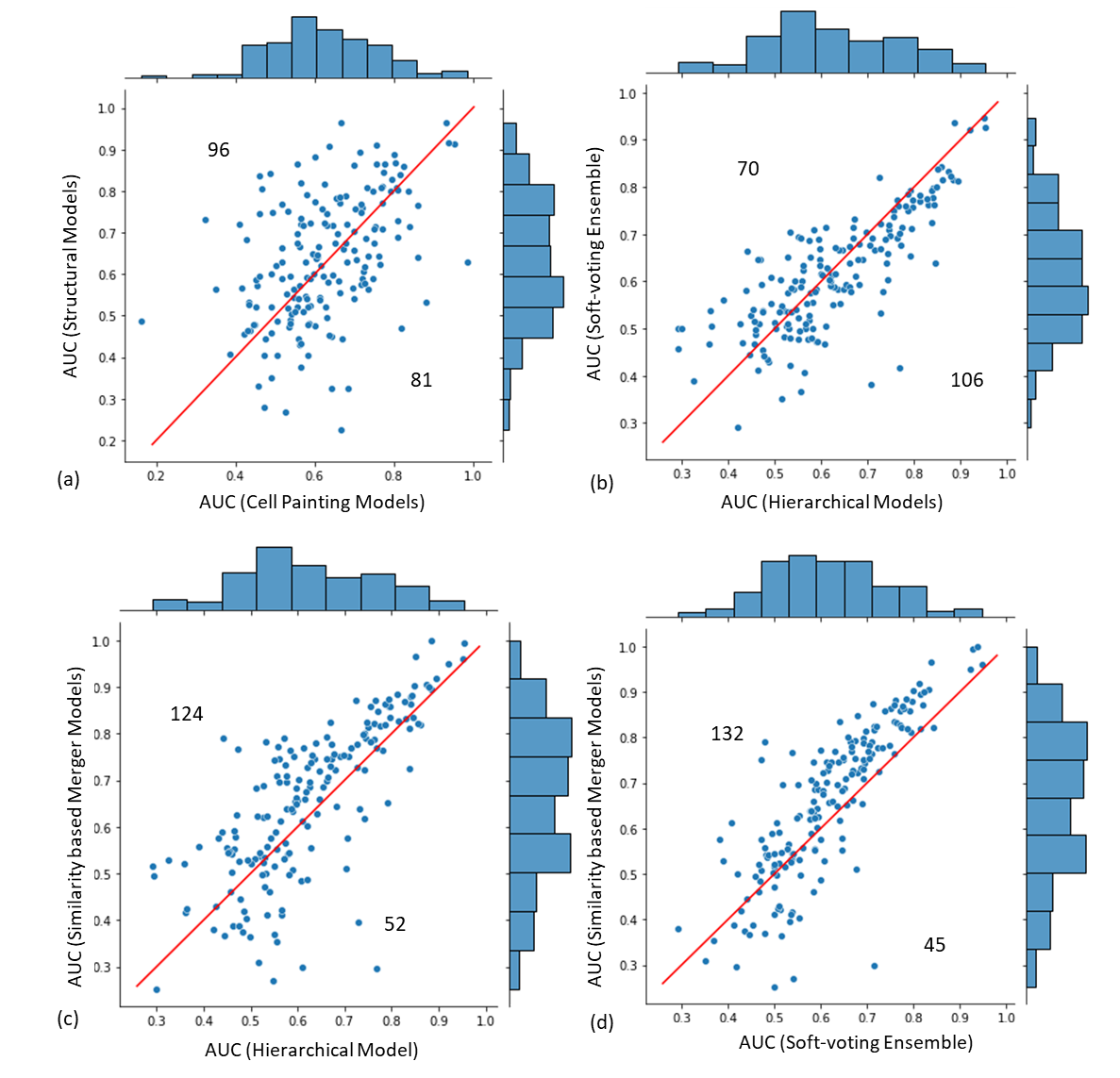


Figure S4: Distribution of AUC Scores for 177 assays used in this study for (a) Cell Painting and Structural Models, (b) Soft-Voting Ensemble and Hierarchical Model, (c) Similarity-based merger model and Hierarchical Model, and (d) Similarity-based merger model and Soft-Voting Ensemble. Any assay above or below the diagonal ($x=y$) line performs better than the other model.


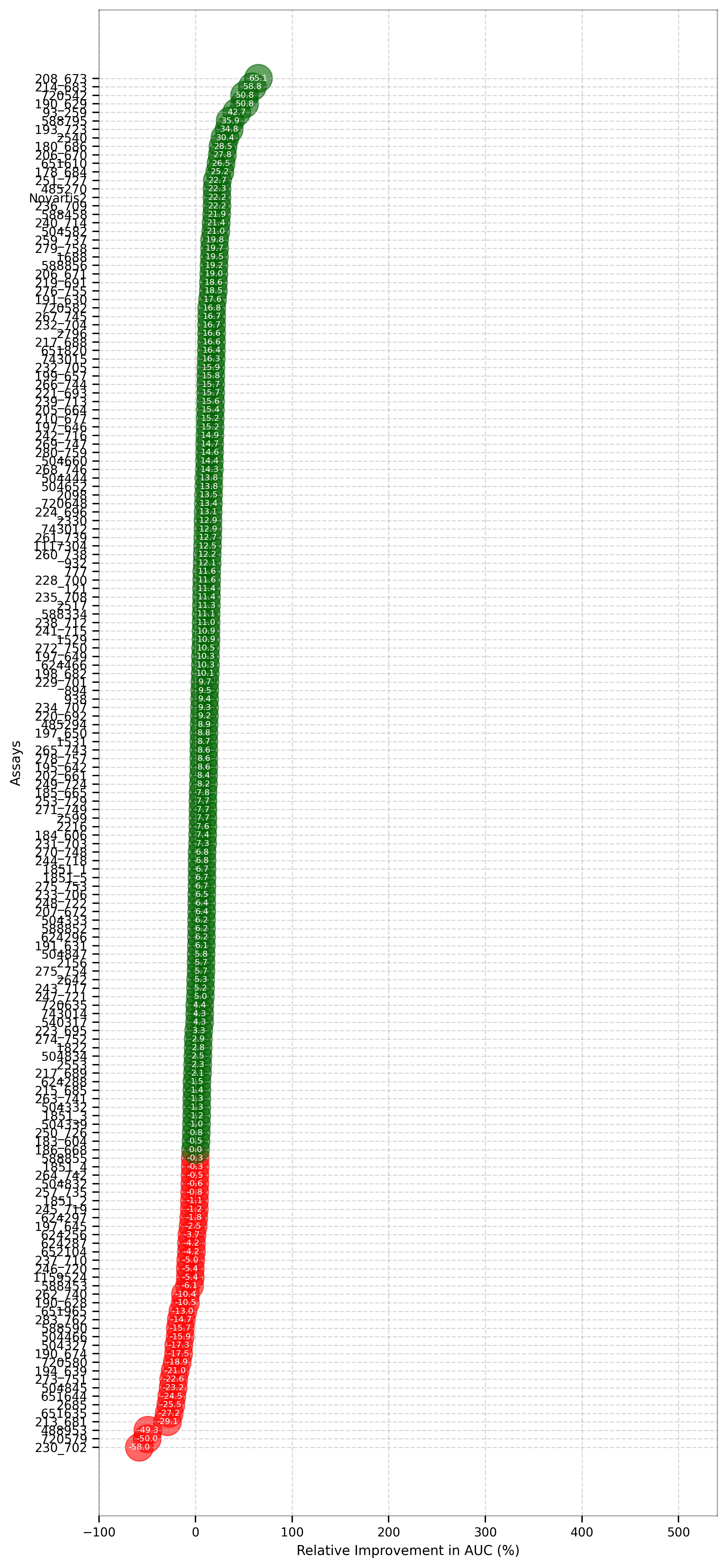


Figure S5: Relative improvement (green) or deterioration (red) in performance on using similarity-based merger models compared to soft-voting ensemble methods over the public dataset comprising 162 assays out of 177 assays where either model performed better than a random classifier (AUC = 0.50)


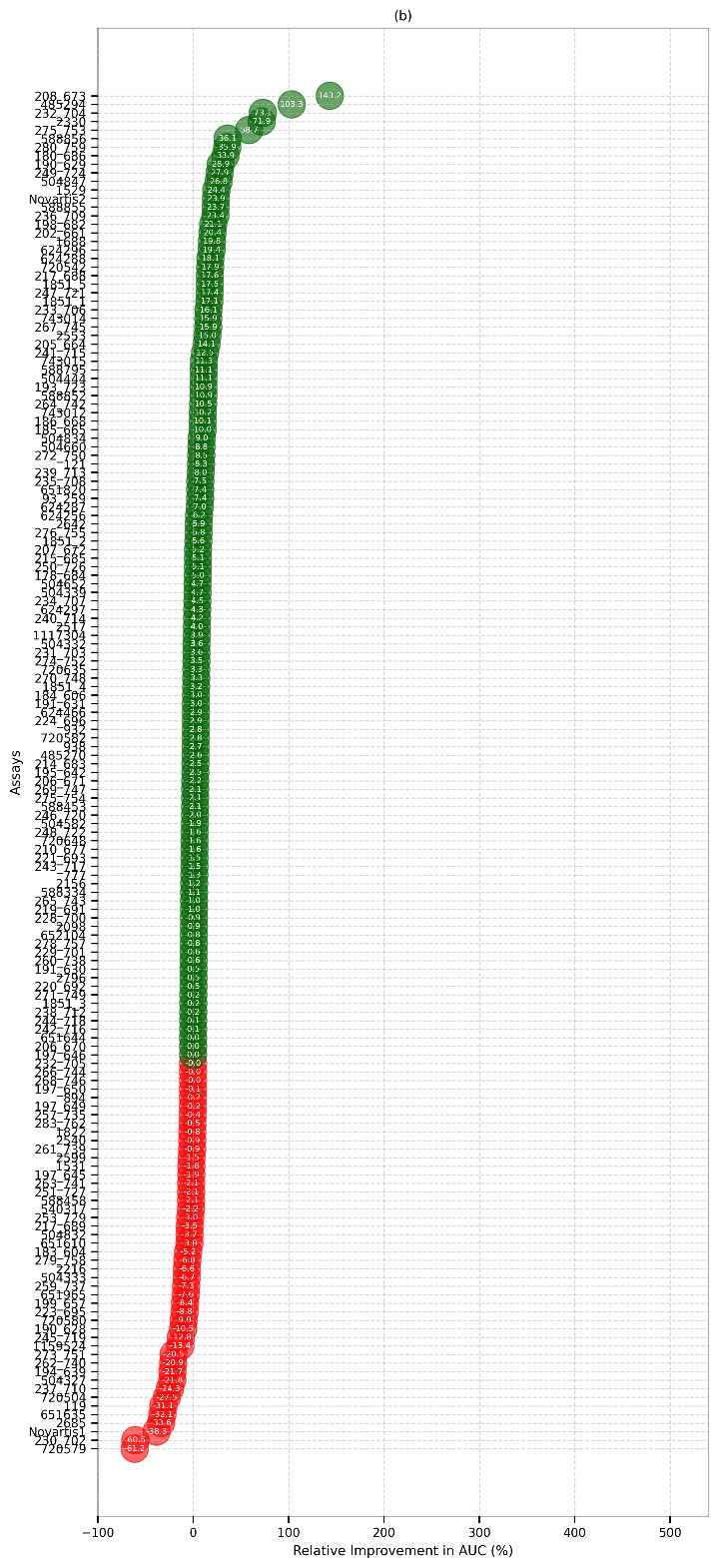

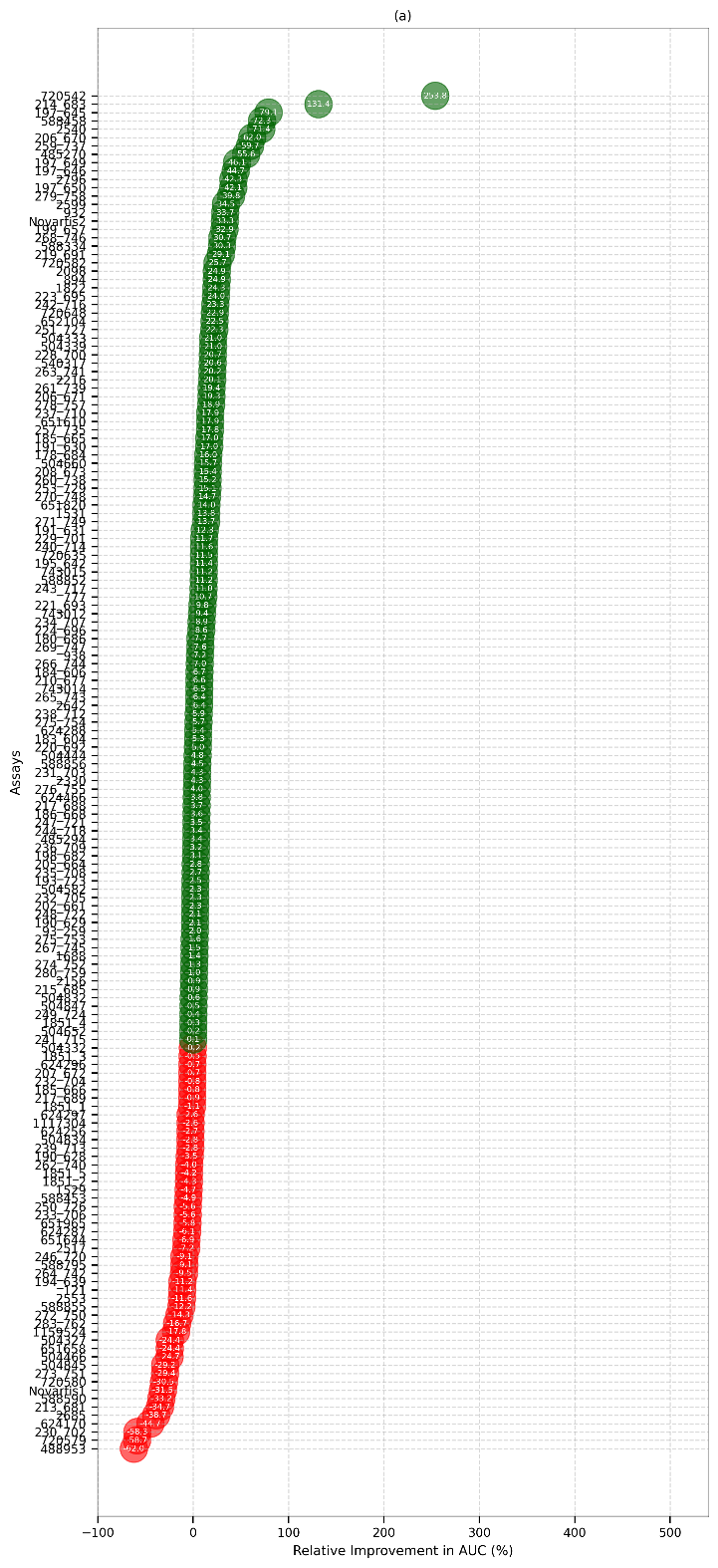

Figure S6: Relative improvement (green) or deterioration (red) in performance on (a) using similarity-based merger models compared to Cell Painting models over the public dataset comprising 163 assays out of 177 assays where either model performed better than a random classifier (AUC = 0.50) , and (b) using similarity-based merger models compared to structural models over the public dataset comprising 159 assays out of 177 assays where either model performed better than a random classifier (AUC = 0.50).


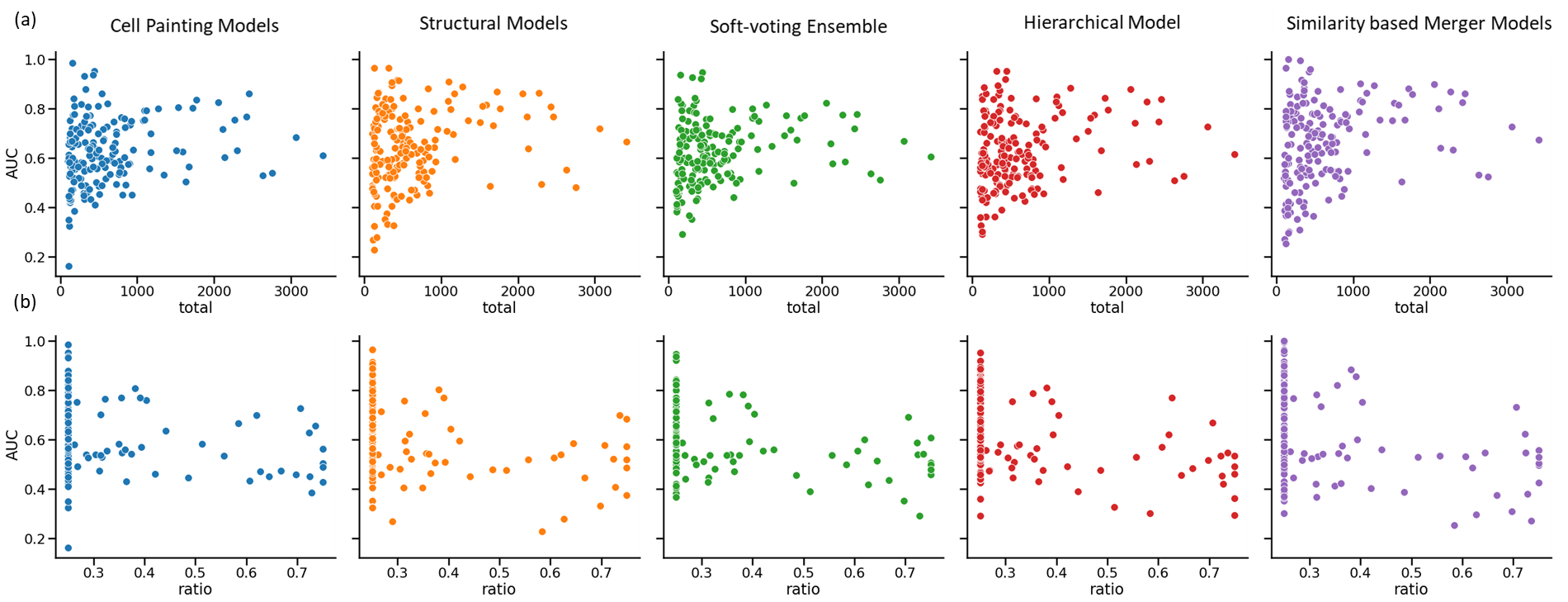


Figure S7: Distribution of AUC scores achieved by individual models for all 177 assays used in this study in relation to (a) the total number of compounds, and (b) the ratio of the number of active compounds to the number of inactive compounds..


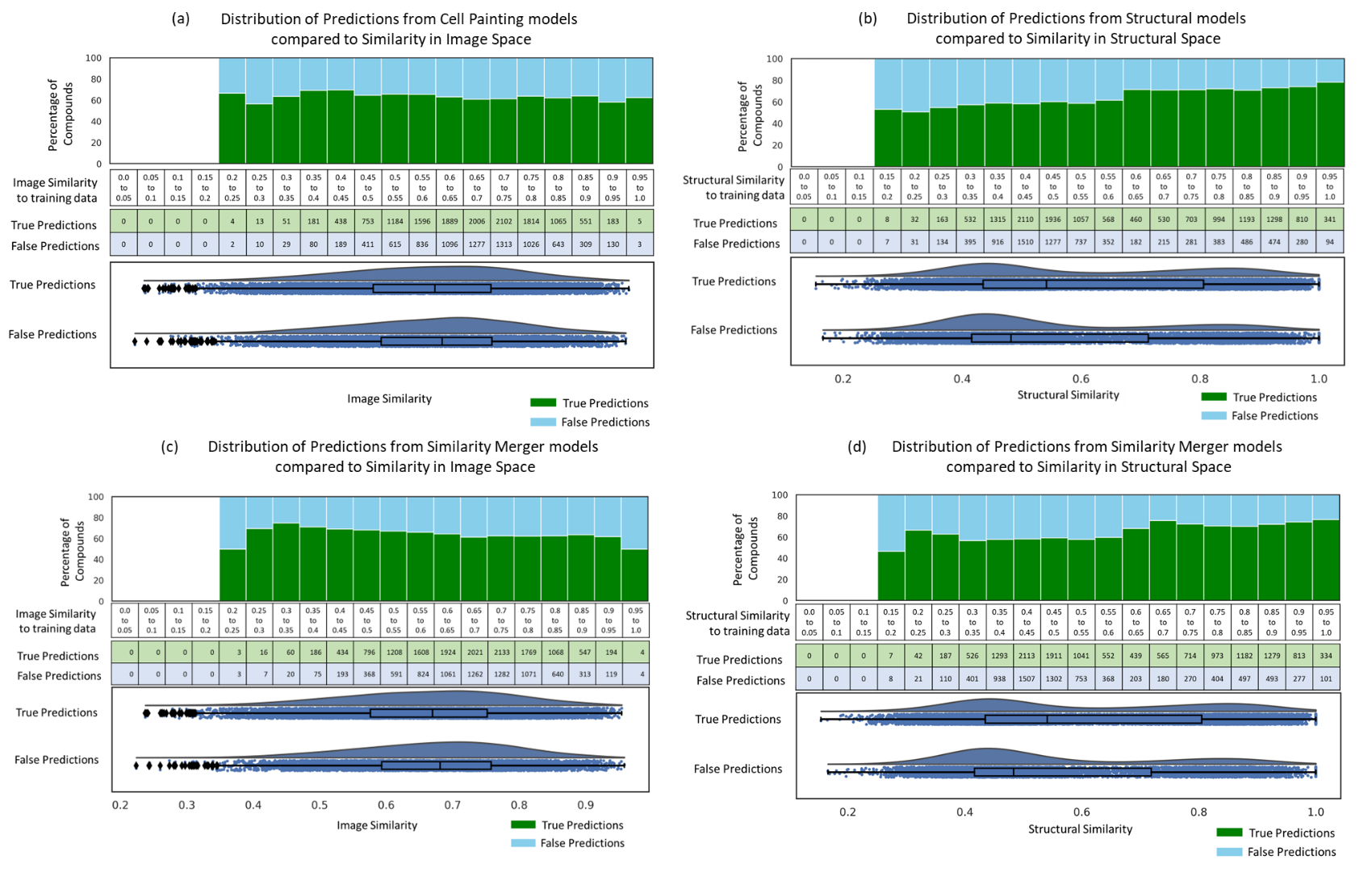


Figure S8: Distribution of True and False Predictions on all compounds in the held-out test for the public dataset over all 177 assays from (a) Cell Painting model compared to the similarity in image space of the respective training set, (b) Structural model compared to the similarity structural space of the respective training set, and the similarity-based merger model compared to (c) the similarity in image space, and (d) the similarity structural space of the respective training set.


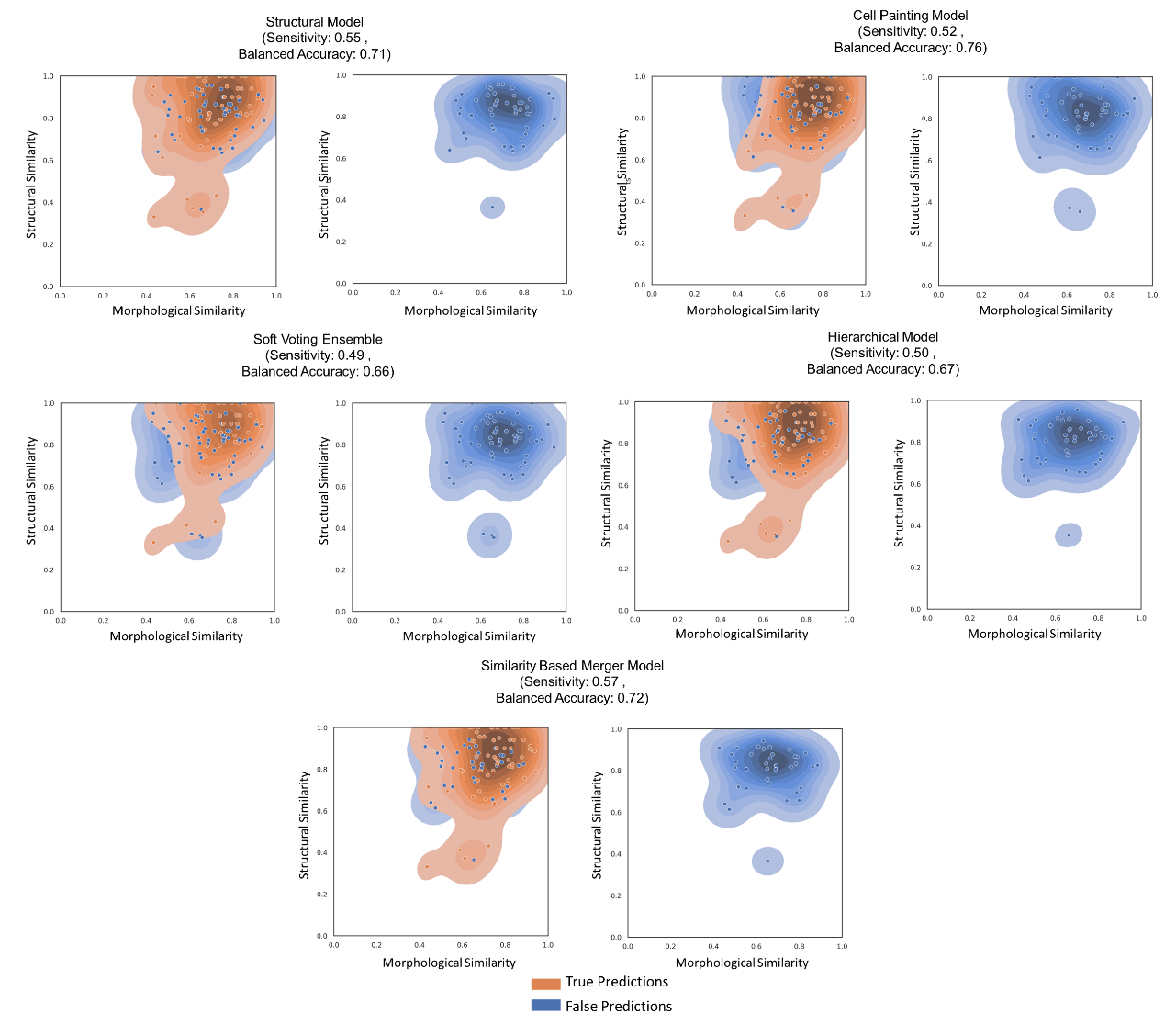


Figure S9: Kernel density estimate (KDE) plot visualising the distribution of True Positives using a continuous probability density curve. The plot shows true positives in the held-out test of the assay 240_714 from the Broad Institute (a fluorescence based biochemical assay) from the individual Cell Painting model, structural model, soft-voting ensemble, hierarchical model, and the similarity-based merger model compared in the space of the similarity in image space and the similarity structural space to the training set used.


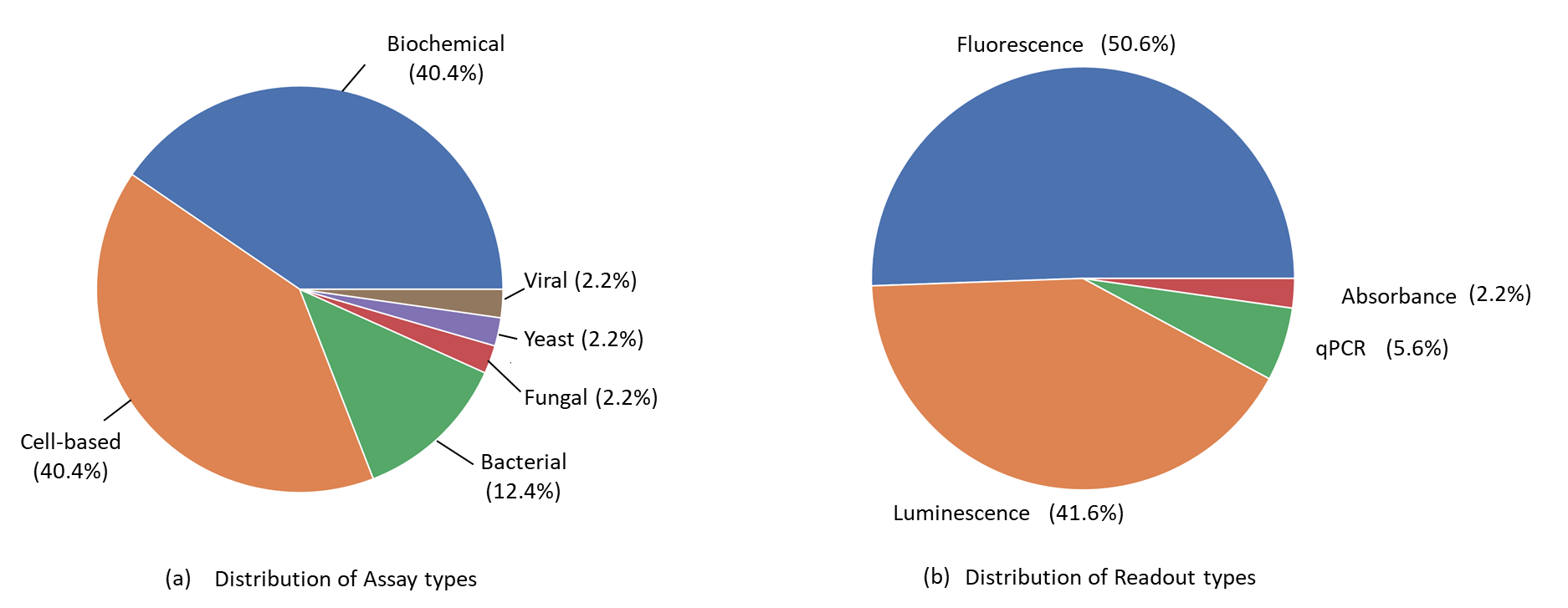


Figure S10: Overview of the assay type and readout types of Broad Institute dataset comprising 89 assays.


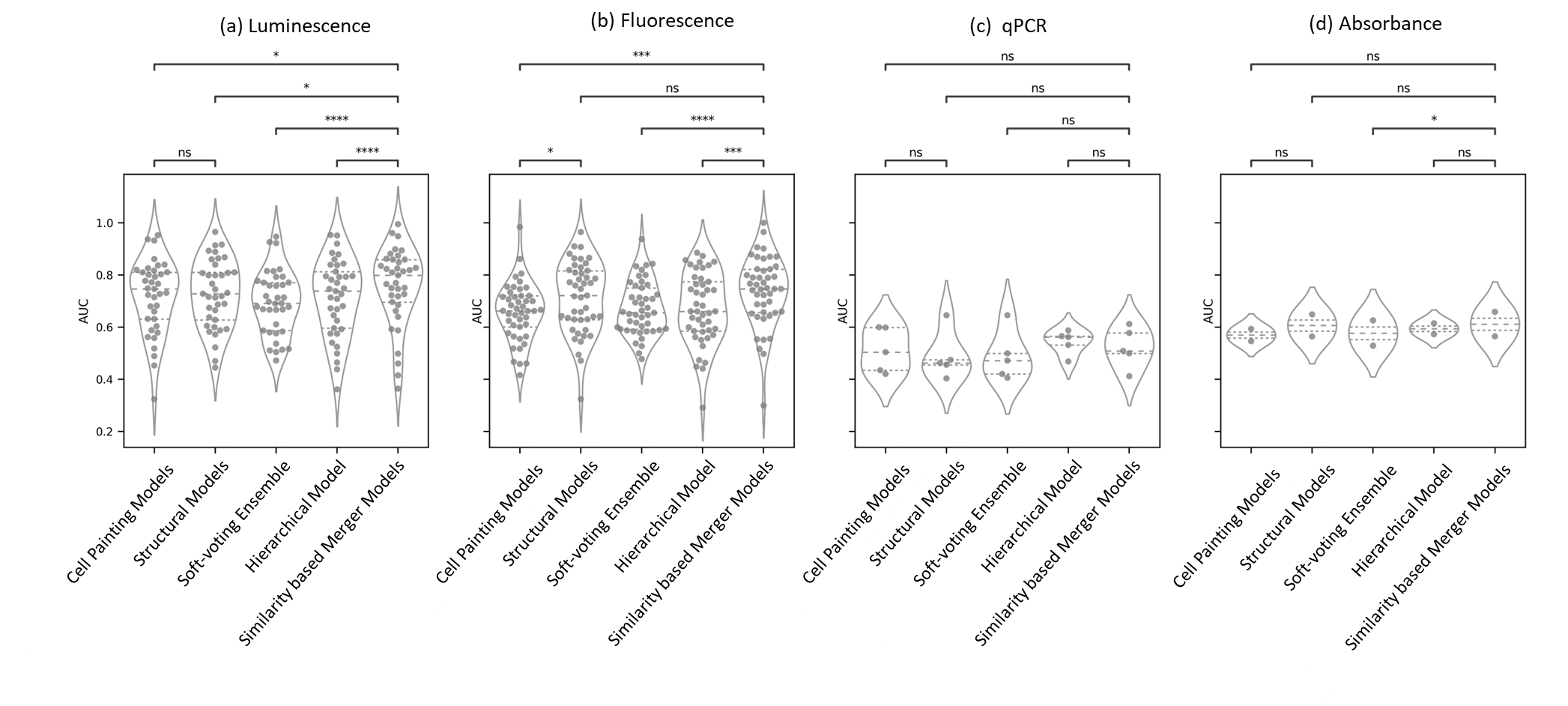


Figure S11: AUC performance of models using Cell Painting, Morgan Fingerprints, baseline models of soft-voting ensemble, hierarchical model, and the similarity-based merger models for 89 assays in the Broad Institute dataset based on readout type.


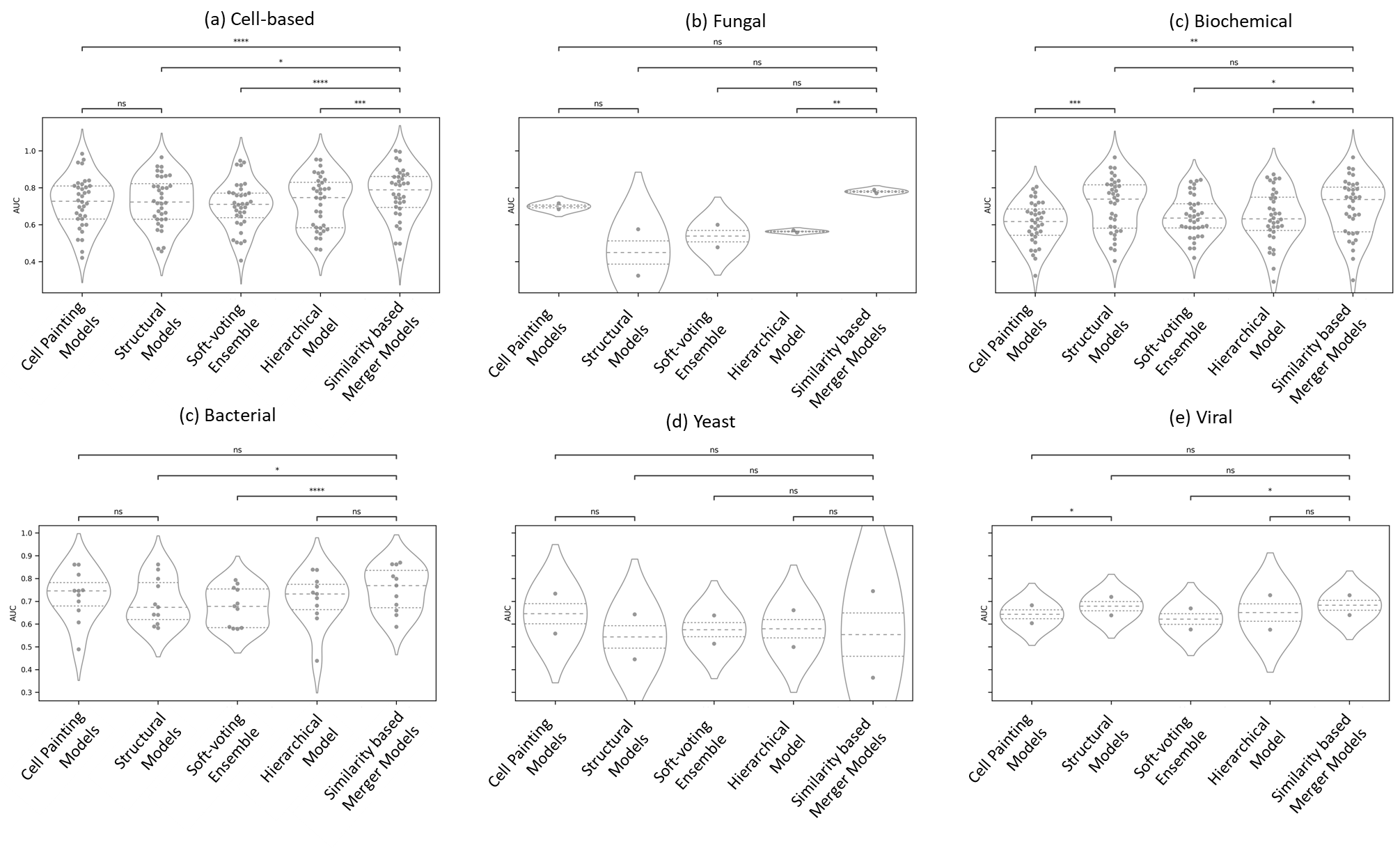


Figure S12: AUC performance of models using Cell Painting, Morgan Fingerprints, baseline models of soft-voting ensemble, hierarchical model, and the similarity-based merger models for 89 assays in the Broad Institute dataset based on assay type.


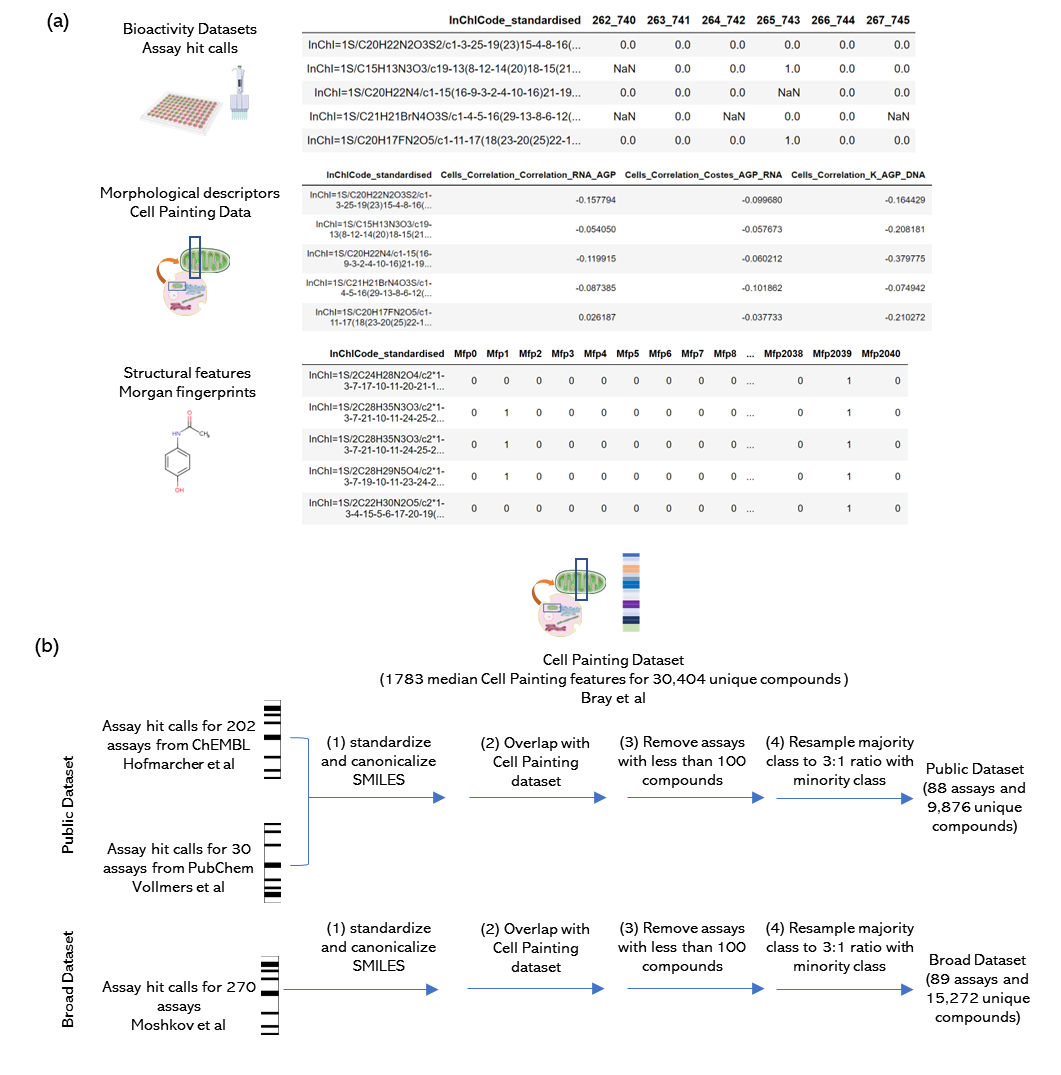


Figure S13: Datasets used in this study: (a) Demonstrative table of data values from bioactivity datasets (binary assay hitcalls), Cell Painting (continuous numerical values) and Morgan fingerprints (binary bit fingerprints) used in this study. (b) Workflow in pre-processing of both bioactivity datasets used in this study, the public dataset comprising 88 assays, and the Broad Institute dataset comprising 89 assays.


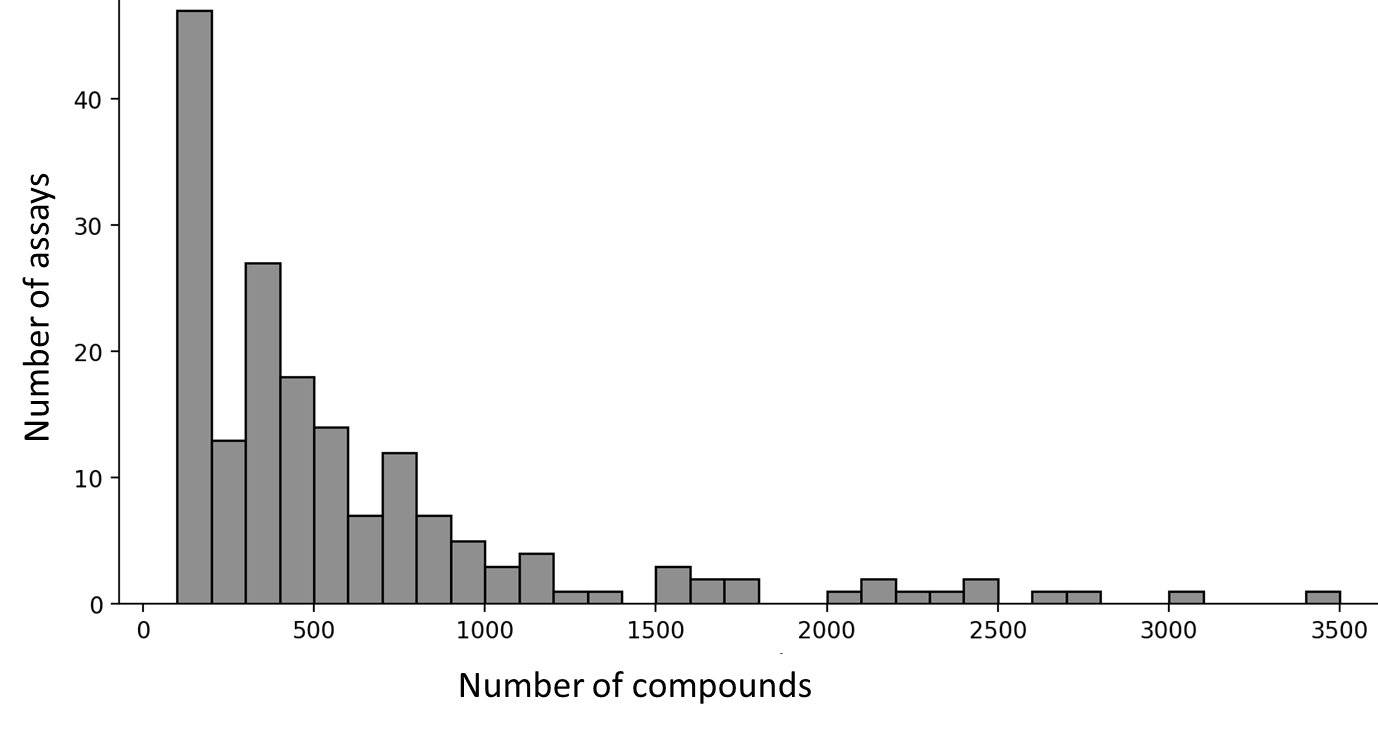


Figure S14: Distribution of number of compounds in each of the 177 assays after under sampling as used in this study
